# Results of the National Breast Cancer Screening Program in Croatia (2006-2016)

**DOI:** 10.3325/cmj.2022.63.326

**Published:** 2022-08

**Authors:** Andrea Šupe Parun, Petra Čukelj, Vanja Tešić, Melita Jelavić, Boris Brkljačić

**Affiliations:** 1 Croatian Institute for Public Health, Zagreb, Croatia; 2 Department of Epidemiology, Dr. Andrija Štampar Institute of Public Health, Zagreb, Croatia; 3 University of Rijeka School of Medicine, Rijeka, Croatia; 4 Department of Diagnostic and Interventional Radiology, Breast Unit, University Hospital Dubrava, University of Zagreb School of Medicine, Zagreb, Croatia

## Abstract

**Aim:**

To assess the uptake of the Croatian National Breast Cancer Screening Program from 2006 to 2016.

**Methods:**

The Croatian National Breast Cancer Screening Program, a biennial program targeting women aged 50-69, started in October 2006. From 2006 to 2016, four cycles were completed. One cycle lasted two years, with the exception of the first cycle, which lasted three years. To determine the number of detected cancers in each cycle, the screening program data were merged with the data of the Croatian National Cancer Registry. Our results were compared with the reference values from the European guidelines for quality assurance in breast cancer screening and diagnosis.

**Results:**

Around 150 000 mammography exams were performed every year. The response rates for cycle 1, cycle 2, cycle 3, and cycle 4 were 63%, 57%, 60%, and 59%, respectively. Further assessment rate was 6.5%. Breast cancer was identified in 5583 women, with 4.8 cancers detected per 1000 mammography exams.

**Conclusion:**

The National Breast Cancer Screening Program in Croatia reached a substantial proportion of the target group. Yet, additional efforts are needed to reach at least 70% of the target population.

Breast cancer (BC) is the most common cancer in women, with 2.26 million patients diagnosed yearly (1). Due to a high incidence and relatively good survival rate, its prevalence is also high. Estimated 7.8 million women (diagnosed in the last 5 years) lived with breast cancer in 2020. BC is a good candidate for screening due to the benefits of early treatment and high cancer prevalence (2).

In Croatia, breast cancer is the leading cancer in women, and the average annual number of diagnosed cases in the 2014-2018 period was 2810. According to the latest data from the Croatian National Cancer Registry, there were 2845 new breast cancer cases (crude incidence rate of 134.7/100,000) in 2018, accounting for 24% of all new cancer cases in women (3). In 2020, 722 women died due to breast cancer (crude mortality rate of 34.7/100,000) (4). According to the European Cancer Information System (ECIS) estimates for female breast cancer in 2020, Croatia is the 19th European (EU-27) country when it comes to breast cancer incidence, and the 16th when it comes to mortality (5).

Chemotherapy, surgery, lymph node analysis, and hormone receptor blocking therapy have improved the survival rate of breast cancer patients (6). Despite these improvements, early breast cancer detection remains vital. Programs for early breast cancer detection using mammography, combined with effective treatment, do not only reduce BC mortality but also positively affect the patients' quality of life (2). The Croatian National BC Screening Program, a biennial program targeting women aged 50-69, started in October 2006.

The European guidelines for quality assurance of mammography-based screening, developed within the Europe Against Cancer program (7), provide necessary framework for reducing the adverse effects of screening, such as diagnostic procedures or treatment resulting from false-positive mammography findings. The Croatian version of the Guidelines was published in 2017, with the goal of setting a framework for further implementation and improvement of the National BC Screening Program in Croatia (8).

Results of the Croatian National Breast Cancer Screening Program are not regularly published. Information on the uptake and number of detected cancers is disseminated to interested stakeholders, but this is the first comprehensive overview of available program indicators. The aim is to present the program results for the 2006-2016 period using available data on performance indicators for breast cancer screening programs.

## MATERIALS AND METHODS

**Figure 1:**
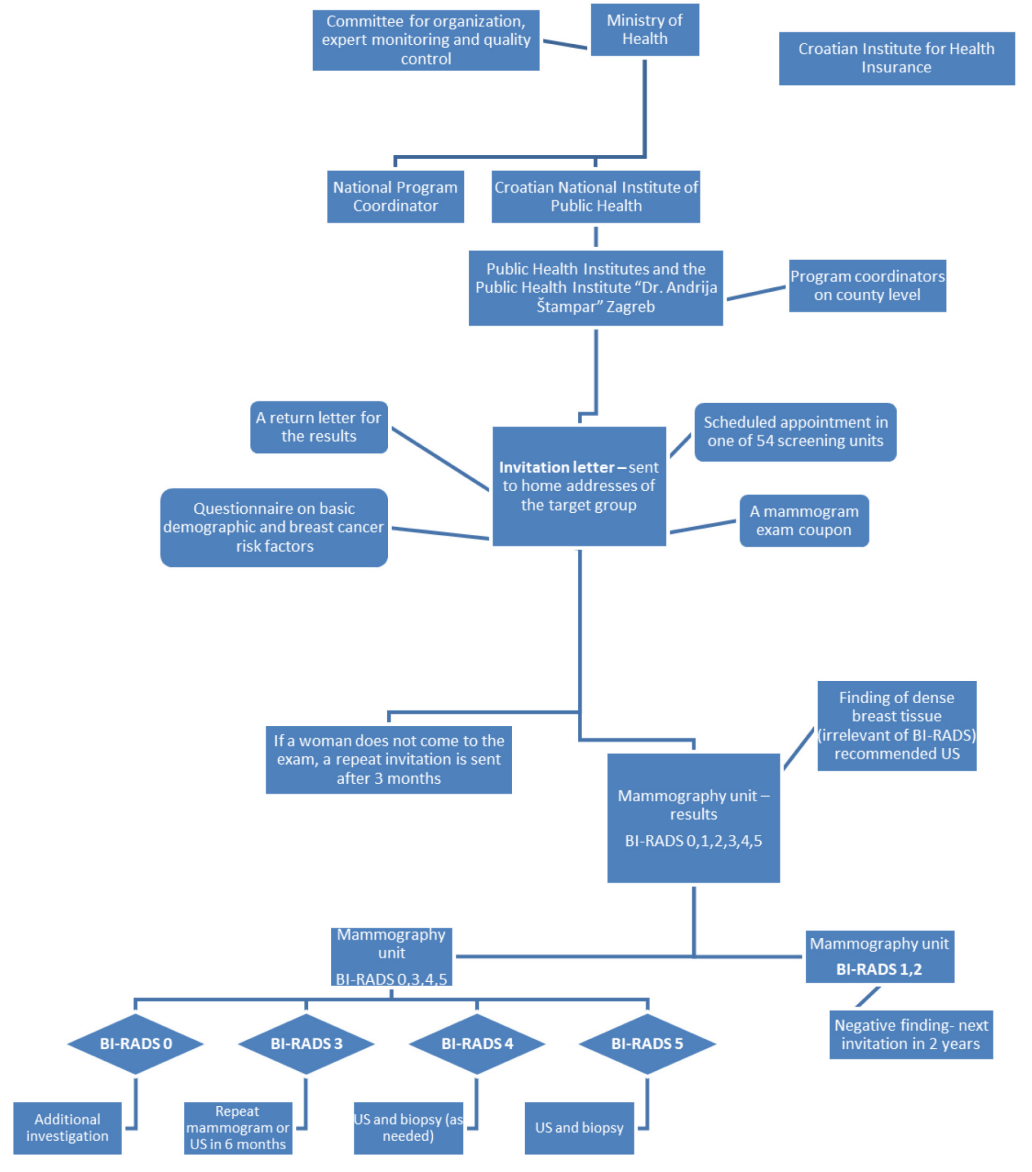
Workflow of the Croatian National Breast Screening Program. BI-RADS – Breast Imaging Reporting and Data System; US –ultrasound.

The Croatian National Breast Cancer Screening Program is organized through public health institutes of 21 Croatian counties and coordinated by the Committee for Organization, Expert Monitoring and Quality Control of the National Breast Cancer Screening Program. The coordinators in each local institute send invitations for a mammography examination, which is conducted by units authorized by the Ministry of Health. Mammography results are categorized from 0-5 according to standardized Breast Imaging Reporting and Data System (BI-RADS) classification (9). Double-reading of the results is conducted by two radiologists. The final result has to be unambiguous, with clear instructions for further steps. It is entered into an application within two weeks, printed out and sent to the participants’ home address together with x-ray images and instructions for further steps. The workflow of the program is shown in Figure 1.

All radiology screening units are obliged to perform secondary assessment of their patients (ultrasound) at their institutions, while all hospitals of category I and II need to perform the necessary core biopsy, or minimally, puncture cytology of the detected lesions.

When each screening cycle is completed, county coordinators compile a written report on the program results on the county level, while the National Program Coordinator compiles a written report on the program results on the national level. Reports are sent quarterly to all members of the Committee. The important indicators available from these reports include the number of patients invited to mammography, number of performed mammographies, cancer distribution according to BI-RADS classification, number of patients in whom BC was diagnosed, stage of cancer spread, and reasons for non-response. The uptake includes the women who received the invitation but replied that they had previously undergone mammography outside of the screening program within a period of one year, or had BC symptoms and had been included in regular mammography follow-up outside of the screening program.

The European guidelines (7) list a number of epidemiologically relevant performance indicators for breast cancer screening, as well as recommended indicators to assess the performance of a breast cancer screening program (10) (Table 1). The only indicator that we were able to determine was the participation rate, and we also calculated further assessment rate as a possible epidemiologically relevant indicator.

**Table 1:** Indicators for the assessment of a breast screening program (9)

Performance indicator	Acceptable level	Desirable level
Participation rate (%)	>70	>75
Technical repeat rate (%)	<3	<1
Recall rate (%)		
initial screening	<7	<5
subsequent-regular screening	<5	<3
Additional imaging rate at the time of screening (%)	<5	<1
Benign to malignant biopsy ratio	≤1:2	≤1:4
Eligible women reinvited within the specified screening interval (%)	>95	100
Eligible women reinvited within the specified screening interval +6 months (%)	>98	100

The analysis was performed with descriptive epidemiology methods. The results were compared with the reference values from the European guidelines for quality assurance in breast cancer screening and diagnosis (7). To determine the number of cancers detected in each cycle, the screening program data were merged with the data of the Croatian National Cancer Registry, a registry collecting data on all cancer cases in Croatia.

## RESULTS

From 2006 to 2016, four screening cycles were completed. The program targeted women aged 50-69. One reference cycle lasted two years, with the exception of the first cycle, which lasted three years.

The first cycle lasted from November 2006 until December 2009. Overall, 665 749 women were invited, and 331 609 mammography exams were conducted. The uptake was 63%. The recommended uptake of 70% was reached in six counties (Bjelovar-Bilogora, Istria, Krapina-Zagorje, Međimurje, Požega-Slavonia, Zadar). A total of 2081 cancers were detected (Table 2).

**Table 2:** Uptake and screening performance data by county in cycle 1 (2006-2009)

County	Mammographies (n)	Uptake (%)	BI-RADS* 0, 4, 5 (n)	BI-RADS 0,4,5 relative to number of mammographies (%)	Cancers detected (n)	Cancers relative to BI-RADS 0, 4, 5 (%)	C/1000 M†
Bjelovar-Bilogora	12 058	88	1027	9.2	72	7.0	6.0
Brod-Posavina	12 420	57	2528	22.2	77	3.0	6.2
Dubrovnik-Neretva	8870	62	934	11.4	74	7.9	8.3
Istria	16 243	76	732	4.9	99	13.5	6.1
Karlovac	9662	58	507	5.7	62	12.2	6.4
Koprivnica-Križevci	10 638	69	2426	24.5	71	2.9	6.7
Krapina-Zagorje	11 589	71	3694	34.2	49	1.3	4.2
Lika-Senj	3452	53	227	7.2	25	11.0	7.2
Međimurje	11 431	86	737	6.9	80	10.9	7.0
Osijek-Baranja	25 769	65	2118	8.9	189	8.9	7.3
Požega-Slavonia	7296	82	290	4.3	40	13.8	5.5
Primorje-Gorski Kotar	22 999	55	1405	6.5	212	15.1	9.2
Sisak-Moslavina	13 711	61	863	6.8	56	6.5	4.1
Split-Dalmatia	28 477	50	2117	8.0	126	6.0	4.4
Šibenik-Knin	9736	62	710	8.0	45	6.3	4.6
Varaždin	13 427	57	770	6.1	96	12.5	7.1
Virovitica-Podravina	6940	66	476	7.3	39	8.2	5.6
Vukovar-Srijem	15 123	65	710	5.0	123	17.3	8.1
Zadar	15 301	82	1326	9.4	99	7.5	6.5
Zagreb County	18 223	51	1242	7.2	96	7.7	5.3
City of Zagreb	58 244	65	5267	9.8	348	6.6	6.0
**Croatia**	**331 609**	**63**	**30 106**	**9.8**	**2081**	**6.9**	**6.3**

*BI-RADS – Breast Imaging Reporting and Data System.

**†**Number of detected cancers per 1000 mammographies.

The second cycle lasted from January 2010 until December 2011. Overall, 642 372 women were invited, and 295 605 mammography exams were conducted. The uptake was 57%. The recommended uptake of 70% was reached in four counties (Bjelovar-Bilogora, Međimurje, Požega-Slavonia, Zadar). A total of 1349 cancers were detected (Table 3).

**Table 3:** Uptake and screening performance data by county in cycle 2 (2010-2011)

County	Mammographies (n)	Uptake (%)	BI-RADS* 0, 4, 5 (n)	BI-RADS 0, 4, 5 relative to number of mammographies (%)	Cancers detected (n)	Cancers relative to BI-RADS 0, 4, 5 (%)	C/1000 M†
Bjelovar-Bilogora	10 705	81	62	0.6	32	51.6	3.0
Brod-Posavina	11 228	51	1686	15.6	56	3.3	5.0
Dubrovnik-Neretva	7147	52	299	4.3	34	11.4	4.8
Istria	17 058	65	606	3.7	87	14.4	5.1
Karlovac	8962	51	310	3.6	31	10.0	3.5
Koprivnica-Križevci	9279	61	612	6.8	48	7.8	5.2
Krapina-Zagorje	10 538	63	2550	24.8	28	1.1	2.7
Lika-Senj	3351	55	99	3.1	10	10.1	3.0
Međimurje	11 285	82	449	4.1	54	12.0	4.8
Osijek-Baranja	21 143	52	1411	6.9	116	8.2	5.5
Požega-Slavonia	6167	71	84	1.4	25	29.8	4.1
Primorje-Gorski Kotar	21 758	51	1184	5.6	127	10.7	5.8
Sisak-Moslavina	11 504	49	268	2.4	33	12.3	2.9
Split-Dalmatia	23 010	35	1078	4.8	108	10.0	4.7
Šibenik-Knin	8006	54	122	1.6	24	19.7	3.0
Varaždin	13 082	56	641	5.1	53	8.3	4.1
Virovitica- Podravina	6191	57	274	4.6	23	8.4	3.7
Vukovar-Srijem	12 705	63	231	1.9	76	32.9	6.0
Zadar	13 159	67	1513	12.0	70	4.6	5.3
Zagreb County	18 720	46	495	2.7	70	14.1	3.7
City of Zagreb	50 607	56	2321	4.8	240	10.3	4.7
**Croatia**	**295 605**	**55**	**16 295**	**5.7**	**1349**	**8.3**	**4.6**

*BI-RADS – Breast Imaging Reporting and Data System.

**†**Number of detected cancers per 1000 mammographies.

The third cycle lasted from January 2012 until May 2014. Overall, 659 975 women were invited, with 262 910 mammography exams conducted. The uptake was 60%. The recommended uptake of 70% was reached in three counties (Bjelovar-Bilogora, Međimurje, Požega-Slavonia). A total of 1269 cancers were detected (Table 4).

**Table 4:** Uptake and screening performance data by county in cycle 3 (2012-2014)

County	Mammographies (n)	Uptake (%)	BI-RADS* 0, 4, 5 (n)	BI-RADS 0, 4, 5 relative to number of mammographies (%)	Cancers detected (n)	Cancers relative to BI-RADS 0, 4, 5 (%)	C/1000 M†
Bjelovar-Bilogora	8632	84	19	0.2	26	136.8	3.0
Brod-Posavina	10 155	58	1046	10.3	40	3.8	3.9
Dubrovnik-Neretva	5598	63	171	3.1	38	22.2	6.8
Istria	13 494	59	397	2.9	63	15.9	4.7
Karlovac	8320	56	146	1.8	38	26.0	4.6
Koprivnica-Križevci	8557	61	382	4.5	46	12.0	5.4
Krapina-Zagorje	9855	64	2038	20.7	37	1.8	3.8
Lika-Senj	2705	50	110	4.1	8	7.3	3.0
Međimurje	10 560	78	436	4.1	53	12.2	5.0
Osijek-Baranja	17 907	56	1224	6.8	89	7.3	5.0
Požega-Slavonia	6021	76	69	1.1	29	42.0	4.8
Primorje-Gorski Kotar	19 548	60	1168	6.0	95	8.1	4.9
Sisak-Moslavina	11 179	55	349	3.1	45	12.9	4.0
Split-Dalmatia	20 548	52	1428	6.9	135	9.5	6.6
Šibenik-Knin	6762	50	31	0.5	33	106.5	4.9
Varaždin	12 843	62	421	3.3	61	14.5	4.7
Virovitica- Podravina	4471	59	208	4.7	23	11.1	5.1
Vukovar-Srijem	10 366	60	246	2.4	62	25.2	6.0
Zadar	12 040	62	1228	10.2	72	5.9	6.0
Zagreb County	18 463	56	615	3.3	74	12.0	4.0
City of Zagreb	44 886	59	2469	5.5	200	8.1	4.5
**Croatia**	**262 910**	**59**	**14 201**	**5.4**	**1 269**	**8.9**	**4.8**

*BI-RADS – Breast Imaging Reporting and Data System.

**†**Number of detected cancers per 1000 mammographies.

The fourth cycle lasted from June 2014 until November 2016. Overall, 622 353 women were invited, with 249 740 mammography exams conducted. The uptake was 59%. The recommended uptake of 70% was reached in three counties (Bjelovar-Bilogora, Međimurje, Požega-Slavonia). A total of 884 BC were detected (Table 5).

**Table 5:** Uptake and screening performance data by county in cycle 4 (2014-2016)

County	Mammographies (n)	Uptake (%)	BI-RADS* 0, 4, 5 (n)	BI-RADS 0, 4, 5 relative to number of mammographies (%)	Cancers detected (n)	Cancers relative to BI-RADS 0, 4, 5 (%)	C/1000 M†
Bjelovar-Bilogora	9016	75	119	1.3	22	18.5	2.4
Brod-Posavina	9018	61	1061	11.8	32	3.0	3.5
Dubrovnik-Neretva	5297	69	183	3.5	21	11.5	4.0
Istria	16 122	65	360	2.2	42	11.7	2.6
Karlovac	7583	51	174	2.3	42	24.1	5.5
Koprivnica-Križevci	7726	58	367	4.8	24	6.5	3.1
Krapina-Zagorje	10 050	63	1643	16.3	28	1.7	2.8
Lika-Senj	2751	50	87	3.2	15	17.2	5.5
Međimurje	10 873	78	872	8.0	35	4.0	3.2
Osijek-Baranja	19 562	62	1086	5.6	73	6.7	3.7
Požega-Slavonia	5549	67	113	2.0	20	17.7	3.6
Primorje-Gorski Kotar	13 202	50	608	4.6	39	6.4	3.0
Sisak-Moslavina	7052	37	314	4.5	16	5.1	2.3
Split-Dalmatia	20 212	51	1120	5.5	85	7.6	4.2
Šibenik-Knin	4764	40	29	0.6	13	44.8	2.7
Varaždin	14 298	66	229	1.6	48	21.0	3.4
Virovitica- Podravina	3611	59	140	3.9	14	10.0	3.9
Vukovar-Srijem	4273	86	185	4.3	13	7.0	3.0
Zadar	11 307	60	897	7.9	49	5.5	4.3
Zagreb County	20 706	60	569	2.7	43	7.6	2.1
City of Zagreb	46 762	60	2564	5.5	210	8.2	4.5
**Croatia**	**249 745**	**59**	**12 721**	**5.1**	**884**	**6.9**	**3.5**

*BI-RADS – Breast Imaging Reporting and Data System.

**†**Number of detected cancers per 1000 mammographies.

**Figure 2:**
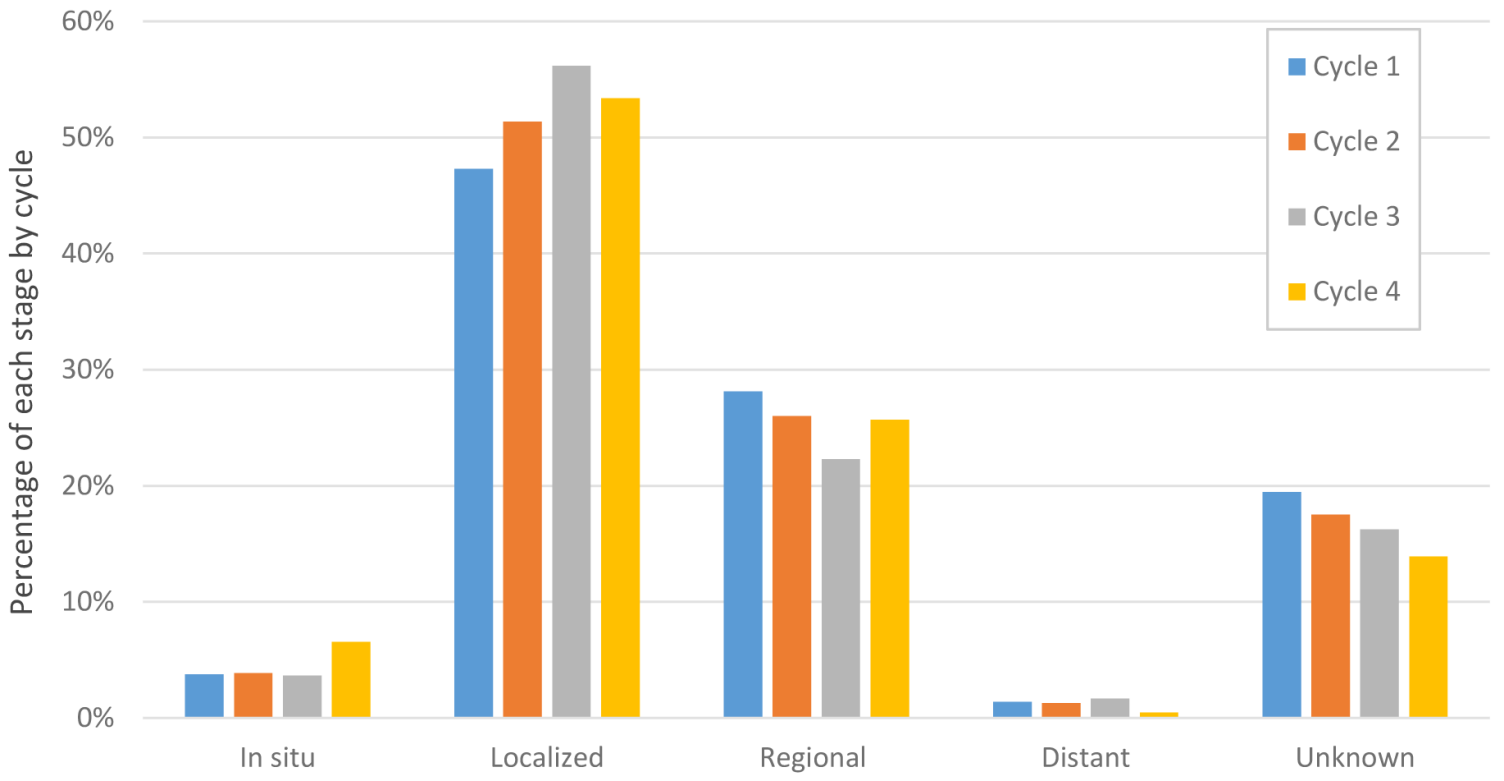
Stage at diagnosis for breast cancers detected by screening per cycle, 2006-2016.

In cycles 1-3, the proportion of localized-stage cancers at presentation increased, while the proportion of cancers with regional spread decreased. In the fourth cycle, we detected fewer BCs, possibly because not all cases from the period were reported to the Cancer Registry. An expected retroactive entry of the data will most likely result in more detected BCs. The percentage of unknown stage at diagnosis varied from 19.5% in cycle 1 to 13.9% in cycle 4 (Figure 2).

## DISCUSSION

Our results show that, 10 years since its implementation, the National Breast Cancer Screening Program in Croatia is sustainable and strong. During the first four cycles, 5583 new breast cancers were detected, 4.8 per 1000 mammographies, which is in line with the expectations. The uptake was around 60% depending on the cycle, which warrants additional efforts should to increase the response.

For such a program to be successful, it is necessary to have adequate technical and human resources, political and public support, and to regularly evaluate program results and make necessary changes (11).

In European countries that have implemented screening programs for 20 years or more, breast cancer mortality rates are decreasing (12). The European Society of Breast Imaging and the majority of national European breast radiology bodies recommend mammography as the method of choice for a breast cancer screening program. The involvement of radiologists to carry out double reading is also recommended (13).

Age-standardized rates of breast cancer incidence in women in Croatia are increasing, similar to other European countries. Age-standardized mortality rates were stable until 2015, with a decrease in the last four years, roughly coinciding with the 10-year anniversary of the implementation of breast cancer screening (4).

The average uptakes of the screening program in cycle 1, cycle 2, cycle 3, and cycle 4 were 63%, 59%, 60%, and 55%, respectively. This is somewhat lower than the 70% uptake recommended by the European guidelines for quality assurance in breast cancer screening and diagnosis (7). The average European participation rate (European total) was 60.2% (14).

The uptake varied substantially between the counties (for example, 37% in Sisak-Moslavina County and 86% in Vukovar-Srijem County during the fourth cycle). Possible reasons include differences in county size and population density, involvement of the local government in the program, and the scope of promotional activities conducted in each county. The availability of mammography units and radiology specialists also differs depending on the county, and can influence the invitation coverage and response rates. Uptake can also be influenced by the degree of involvement of primary care physicians and patronage nurses. A positive example of this is Međimurje County, with good organization, regular promotional activities, and excellent cooperation of all stakeholders in the program, all of which lead to one of the highest uptakes in Croatia in all four cycles.

The City of Zagreb had an average uptake in all four cycles. We believe that in the City of Zagreb a considerable number of women undergo mammography exams in private clinics, and as a part of regular physical exams organized by their employer. Unfortunately, we do not receive data from these medical institutions.

In the first three cycles, the expected number of detected cancers per 1000 mammography exams was reached, with 5-6 detected cancers per 1000 exams. This number was not reached in the fourth cycle, probably due to data quality, or because not all cancers were recorded in the Croatian National Cancer Registry, especially for later years.

Overall, 5583 breast cancers were detected, translating to 4.8 detected breast cancer cases (both invasive and in situ) per 1000 mammographies for these four cycles. The rate was higher in the initial cycle, 6.3/1000, while in the subsequent cycles it was 4.3/1000. In comparison, an average rate for the EU Member States (13) was 6.2 per 1000 women screened (from 2.3 in Portugal, Alentejo to 10.2 in Wales); 7.2/1000 for the initial tests and 5.6/1000 for subsequent cycles. The average further assessment rate in the first four cycles was 6.5%, while the European total was 5.2% (14).

Age-standardized mortality of breast cancer in Croatia decreased by 23% between 2007 (35.9/100,000) and 2020 (27.5/100,000, 2011 Census standard population) (4). Since the only way to determine the effect of a specific breast cancer screening program on breast cancer mortality is to conduct a randomized controlled trial, it is not possible to know if, and to what extent, this finding is the result of the screening, as the improvements in treatment have certainly played a role. However, breast cancer screening can lead to a 30% reduction in breast cancer mortality (15), and although the uptake in Croatia is somewhat lower than prescribed by the screening guidelines, it is presumably still high enough to yield a beneficial effect.

Mammography screening allowed a detection of an increased number of cancers at an early, localized stage, and of a decreased number of cancers with regional and distant metastasis (16,17). These findings indicate that some of the goals of screening program have been achieved. An early diagnosis and early treatment are essential to improve the outcomes of this disease. Therefore, screening programs that allow for early disease detection are a vital public health strategy.

There are some limitations to this study. First, data on almost all of the indicators mentioned in the European guidelines are not routinely collected as part of the program, which limits the possibility of adequate evaluation of the program results. The data on detected cancers and stage at diagnosis were collected by merging the screening database with the Cancer Registry database, and the obtained number of cancer diagnoses was lower than expected when compared with the EU average. We hope that an improved IT infrastructure will enable the follow-up of each woman throughout the whole screening process, from invitation to cancer diagnosis and treatment. Another limitation is the difference in the duration and circumstances surrounding each of the cycles. Due to problems concerning public procurement, the first cycle lasted for 3 years, which limits the comparison with subsequent cycles. Although more breast cancer cases are expected to be diagnosed in the initial cycle, we believe that in this case the difference is in part due to differences in cycle duration.

Despite some difficulties, we are satisfied with the current development of the program. All the studied parameters have significantly improved, which indicates that the program led to true benefits for the patients. These first results show an average screening uptake and an average number of detected cancers. Additional efforts are necessary to obtain other relevant performance indicators and to further improve the uptake.
